# Systematic Drug Repositioning Based on Clinical Side-Effects

**DOI:** 10.1371/journal.pone.0028025

**Published:** 2011-12-21

**Authors:** Lun Yang, Pankaj Agarwal

**Affiliations:** Computational Biology, Quantitative Sciences, Medicines Discovery and Development, GlaxoSmithKline, Philadelphia, Pennsylvania, United States of America; Semmelweis University, Hungary

## Abstract

Drug repositioning helps fully explore indications for marketed drugs and clinical candidates. Here we show that the clinical side-effects (SEs) provide a human phenotypic profile for the drug, and this profile can suggest additional disease indications. We extracted 3,175 SE-disease relationships by combining the SE-drug relationships from drug labels and the drug-disease relationships from PharmGKB. Many relationships provide explicit repositioning hypotheses, such as drugs causing hypoglycemia are potential candidates for diabetes. We built Naïve Bayes models to predict indications for 145 diseases using the SEs as features. The AUC was above 0.8 in 92% of these models. The method was extended to predict indications for clinical compounds, 36% of the models achieved AUC above 0.7. This suggests that closer attention should be paid to the SEs observed in trials not just to evaluate the harmful effects, but also to rationally explore the repositioning potential based on this “clinical phenotypic assay”.

## Introduction

Repositioning helps fully explore the indications of marketed drugs and clinical candidates [1]; however, most successful stories of repositioning are based on serendipity and not systematic analysis [2]. *In silico* methodologies have helped in mining the drug's *off-target* effects [3], [4], [5], [6], [7], [8], *off-system* effects (such as, off-target related gene expression perturbation or downstream pathways) [9], [10], [11], [12], [13] and *off-phenotypes* (i.e. adverse drug reactions [14], [15] or new indication) providing new hypotheses to reposition the drug. These strategies focus primarily on using preclinical information. Unfortunately, clinical therapeutic effects are not always consistent with preclinical outcomes [16].

Recently, a systematic analysis observed that phenotypic screening exceeded target-based approaches in discovering first-in-class small-molecule drugs [17]. Clinical phenotypic information comes from actual patient data, which mimics a phenotypic “screen” of the drug effects on human, and can directly help rational drug repositioning. For example, Chiang and Butte suggested new indications for a drug based on its existing therapeutic effect [18]. In our study, however, we utilize the rich information from the clinical side-effects (SEs), which are usually regarded only as unwanted effects to suggest new indications for a drug. For instance, *hypotension* is an unfavorable SE of some drugs. However, those drugs may also act as anti-hypertensives, if we utilize this SE by controlling the dosing, improving the formulation and choosing the sub-population etc.

The rationale for this strategy is that SEs and indications are both measurable behavioral or physiological changes in response to the treatment, and if drugs treating a disease share the same SE, there might be some underlying mechanism-of-action (MOA) linking this disease and the SE. The SE may thus serve as a phenotypic “biomarker” for this disease. Furthermore, both therapeutic and side effects are observations on human subjects, as opposed to animal models, so there is less of a translational issue. The methodology of Drug Repositioning based on the Side-Effectome (DRoSEf) is discussed in this study. The basic hypothesis is that if the SEs associated with a drug D are also induced by many of the drugs treating disease X, then drug D should be evaluated as a candidate for treating disease X. We constructed a database of disease-SE associations from drug-SE data extracted from drug labels by SIDER and drug-disease relationships from PharmGKB (**Table S1**). Researchers, who observe an unexpected effect in their clinical trial can query the database for other diseases associated with this phenotype. This would suggest alternative indications for the drug. Using this approach, we predict new indications for marketed drugs. In addition, we built QSAR models to predict side effects based on the compound structure. For 4,200 candidate drugs with no available clinical SE information, we were able to combine the above QSAR models with the SE-disease models to predict new indications.

## Results

### Identification of the disease-side effect associations

**Figure 1:**
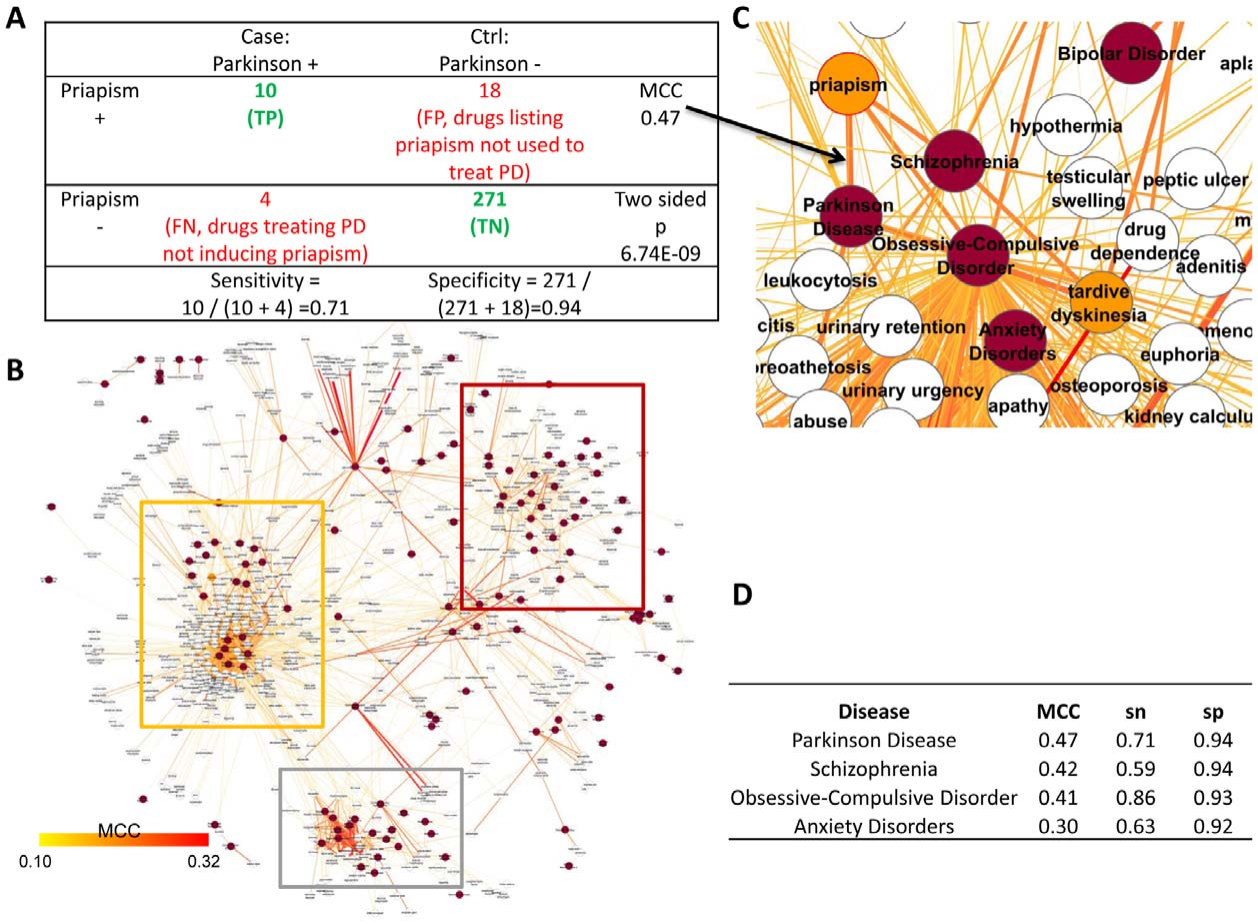
Constructing and visualization of the disease-SE associations. a) Confusion matrix of using SE *priapism* to predict Parkinson Disease. PD: Parkinson Disease; MCC: Matthews correlation coefficient; TP, FP, TN and FN stand for true positives, false positives, true negatives and false negatives respectively. This confusion matrix represents one disease-SE pair. b) The overall layout of the disease-SE network. Diseases and SEs are shown in red and white circles respectively. The edge color and the width indicate the association strength as measured by MCC. The neuropsychiatric, neoplasm, circulatory- system disease dominated clusters are highlighted in yellow, red and grey rectangles respectively. c) Neuropsychiatric disease-dominated cluster. The SE *tardive dyskinesia* and *priapism* is highlighted in orange. The MCC for PD-*priapism* pair is 0.47 according to the confusion matrix in a) and is visualized as an orange line (see black arrow from a) to c)) . d) Sample MCC, sensitivity and specificity measures for using *priapism* to predict diseases. For example, 86% of the drugs that treat obsessive-compulsive disorder (OCD) list *priapism* as a side effect; whereas only 7% (1-sp) of the drugs not reported to treat OCD list this SE.

Both disease-drug associations and drug-SE associations are required to infer disease-SE associations. We extracted the indications of drugs from PharmGKB to provide the disease-drug associations [19]. The SEs printed on the drug label provide consistent and reliable data as these are summarized from large clinical trials, and the drug label is approved and standardized by regulatory agencies. The SIDER database [4], which has been used to predict drug off-targets provides a mapping extracted from drug labels of 888 approved drugs to 584 side effects. These 888 drugs map to 303 drugs and 145 diseases in PharmGKB. We used the binary fact of the SE's presence on the drug label as listed in SIDER. Similar to generating gene-SE associations in ref [20], we inferred disease-SE associations by counting the number of the drugs listing or not listing a SE when indicated or not indicated for a disease, generating a confusion matrix as shown in **Fig. 1A**. The association strength of a disease-SE pair is measured using multiple criteria, including the Matthews correlation coefficient (MCC), sensitivity (sn) and specificity (sp). We computed 84,680 confusion matrices for each pair of 145 diseases and 584 SEs. 3,175 (3.75%) of these associations (**Table S1**) were considered possibly informative (using multiple criteria as described in **Methods**).

We investigated a few of the 3,175 associations to understand what these associations implied and how they could be used to suggest new indications. Some of the associations have an explicit explanation based on the current knowledge of the MOA (**Table 1**). The SE *positive Antinuclear Antibodies (ANA)*indicates the presence of autoimmune antibodies and appears to be associated with stroke. It is the SE shared by drugs treating stroke, mainly ticlopidine and several angiotensin-converting enzyme (ACE) inhibitors. Stroke itself, is associated with severe immune suppression [21]. Thus, conceivably drugs that are associated with increasing immune response in terms of *positive ANA* may help stroke patients, though of course an autoimmune response is not desirable. Overall, 50% of the drugs treating stroke list this SE, whereas only 2% of the drugs not indicated for stroke list *positive ANA* as a SE. This 2% (often termed false positives) includes several statins and ramipril (**Table 1**). Several statins are associated with *positive ANA*, but are not indicated for stroke. However, a meta-analysis of 120,000 patients across 42 trials showed that statin therapy provides protection for all-cause mortality and nonhemorrhagic strokes [22]. Ramipril, which also lists *positive ANA* as a SE, showed a 32% risk reduction for stroke [23]. DRoSEf is suggesting that the immune related SEs of these drugs directly indicate their use for stroke, and this has also recently been recognized experimentally [23].

**Table 1:** Some of the associations from the disease-SE network.

Disease Class	Disease	Side Effect	MCC	sn	sp	p value	Predictions (False Positive Drugs)^a^
**Circulation System**	Stroke	Positive ANA	0.46	0.47	0.98	1.8E-15	statins, ramipril
**Other**	Transplantation	Cytomegalovirus infection	0.75	0.75	0.99	3.5E-06	methotrexate
**Metabolite disease**	Diabetes Mellitus	Porphyria	0.44	0.50	0.98	8.8E-06	valproic acid, pyrazinamide, naproxen, estradiol
**Psychiatric disease**	Depressive Disorder	Delusions	0.46	1.00	0.91	1.1E-08	cabergoline, memantine, pergolide
**Psychiatric disease**	Depressive Disorder	Hyperacusis	0.55	0.88	0.96	9.0E-09	phenytoin, modafinil
**Neoplasms**	Neoplasms	Constitutional symptoms	0.50	0.56	0.94	2.6E-18	nevirapine

a Drugs not listed treating disease (2^nd^ column) but listed the SE (3^rd^ column).

Cytomegalovirus infection is a sign of a weakened immune system [24]. Drugs that reduce immune response are often used to prevent transplant rejection, thus drugs that list increased *cytomegalovirus* (*CMV*) *infections* as a SE may be good candidates for treating transplant patients. Methotrexate, an antineoplastic drug lists *CMV infections* as a SE. As a dihydrofolic acid reductase inhibitor, it is officially used as an antineoplastic, but has been reported for the off-label use of preventing transplant rejection [25].

DRoSEf suggests that drugs that list *porphyria* as an SE may act as antidiabetics. In a study of 328 Swedish patients with *porphyria*, the 16 patients that developed diabetes all had their *porphyria* symptoms resolved [26]. Valproic acid, pyrazinamide, naproxen, and estradiol all list *porphyria* as a SE but are not indicated for diabetes. Valproic acid is an anticonvulsant and a recent study found it effective in lowering blood glucose levels in *Wfs1* knockout mice [27]. Pyrazinamide is an anti-tuberculosis agent, and type II diabetes is a known risk factor for tuberculosis [28]. In mice, naproxen is used as a tool to delay or prevent the development of type II diabetes from a pre-diabetic condition [29]. In a double-blinded, randomized placebo controlled clinical trial on women with type II diabetes, oral estradiol significantly decreased fasting glucose [30].

Drugs that list *delusions* as a side effect may help with depression. Cabergoline, an ergot derivative that causes *delusions*, is a dopamine agonist that has an antidepressant-like property [31]. The dopamine receptor agonist pergolide has shown antidepressant effects in Parkinson patients [32]. *Hyperacusis* is a medical condition associated with hypersensitivity to certain frequency ranges of sounds. Phenytoin is a known anticonvulsant with *hyperacusis* as a listed side effect, and DRoSEf suggests a potential utility for treating depression. In fact, a small clinical trial found equivalent therapeutic effects between phenytoin and fluoxetine in treating depression [33]. Modafinil is a drug for narcolepsy and is also potentially effective in combination with fluoxetine to treat depression [34].

*Constitutional symptoms* are a listed SE for many antineoplasm drugs. An anti-HIV drug nevirapine also lists *constitutional symptoms* as a SE. Nevirapine has previously been suggested as a treatment for human hormone-refractory prostate carcinoma [35].

In fact, 27% of the “false positive” drugs-disease association (**Table S2**) suggested by DRoSEf have at least one article in PubMed of publication type “clinical trial” with the drug name mentioned, and the disease a major subject heading for that article. Still not all 3,175 associations have an obvious MOA explanation based on current knowledge. We thus include all of the associations in **Table S1** and look forward to further experiments, and analysis. Based on these 3,175 associations, we built Naïve Bayes models to predict the 145 indication endpoints using their associated SEs as the features. The average AUCs of 10-fold cross validations for each of the 145 disease were calculated using Weka [36]. 92% of the AUCs were above 0.8 (**Table S3**) suggesting that multiple SEs can be used to predict indications.

### Visualization of the disease-SE associations

Based on these 3,175 associations, a disease-SE network was constructed (**Fig. 1B**). Diseases that share similar SEs tend to cluster with each other. The diseases are grouped into three clusters dominated by neuropsychiatric diseases (**Fig. 1C**), circulatory system diseases, and neoplasms (**Figure S1**) as visualized using Cytoscape [37]. The neuropsychiatric disease-dominated cluster (**Fig. 1C**) shares SEs, such as *tardive dyskinesia*, an involuntary movement SE associated with long term dosing or high doses of antipsychotics [38]. Other SEs, such as *priapism*, a painful medical condition in which the erect penis or clitoris does not return to flaccid state [39], is also shared by four neuropsychiatric diseases (**Fig. 1D**). For instance, the connection between *priapism* and OCD suggests repositioning opportunities of *priapism* compounds to OCD (**Text S1**).

### DRoSEf for compounds in clinical trials

The prior analysis requires knowing the SEs from a drug's label before we predict new indications. For clinical candidates whose SE information is unknown, we predicted their SEs based on the compound structure and then predicted new indications based on those SEs. We hypothesized that such a prediction “chain” would provide mechanistic explanations of the compound's new indication based on the disease-SE association and the structural information of the compound. We extracted all small molecules from Genego® MetaBase along with their disease indications [40]. This provided molecules in clinical trials in addition to the 888 SIDER drugs. These 4,200 molecules are indicated for at least one of 101 diseases from the 145 disease set. MetaBase also uses MeSH disease terms, thus making comparisons to the MeSH indications from PharmGKB straightforward.

**Figure 2:**
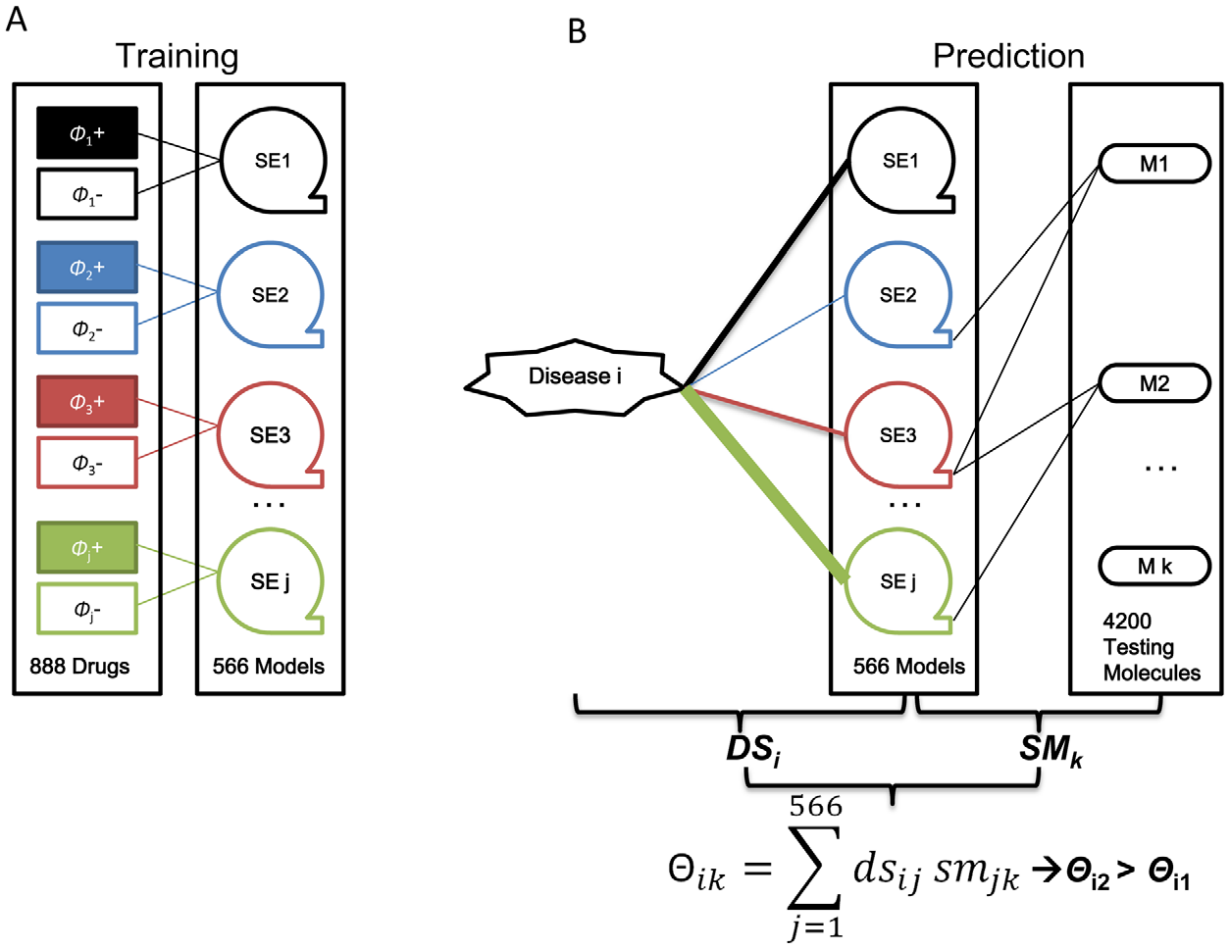
A schematic figure of mapping drug structure to side effect and then onto a disease indication. a) Train the 566 SE models. For SE_j_, the 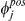 (+) and 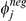 (−) were recruited from 888 SIDER molecules. b) The disease_i_-molecule_k_ association (Θ_ik_) was calculated as the dot product value of the disease-SE association vector (DS) and SE-molecule association vector (SM). The binary SE-molecule (SM) association was calculated from QSAR models. The width of the colored lines indicates the weights of the disease-SE associations. As an example, Θ_i2_ is more than Θ_i1_ as the association of side effect *j* in green to disease *i* is stronger.

DRoSEf requires the side-effect profile for each molecule to predict new indications. However, such information is difficult to obtain for most of the 4,200 molecules because these are generally clinical candidates without FDA approved drug labels, and have little or no SEs published from their clinical trials in a standardized way. Quantitative structure-activity relationship (QSAR) models have been used to predict target binding of the ligand [41]. We hypothesized that QSAR models could also be used to predict SEs. We are mapping compound structure to possible SEs and then onto a disease indication. For side effect *j* (SE_j_), we recruited the positive set (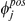 , drugs listing SE_j_) and the negative set (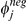, drugs not reported to induce SE_j_) from the 888 SIDER drug set (**Fig. 2A**). For 566 of the 584 SEs, we successfully trained QSAR models (18 of them failed). Then we used these 566 QSAR models to predict the SEs of 4,200 molecules from MetaBase (**Fig. 2B**, **Methods S1**).

The ROC curves of the prediction performance for 101 disease endpoints are shown in **Figure S2**, and their AUCs are summarized in **Table S4**. Some of the disease endpoints had only a few positive drugs from the MetaBase set, and their AUC value might not accurately reflect the true performance. We, therefore, focus on the diseases that have more than 30 compounds with that specific indication in MetaBase. **Table 2** lists the diseases with AUC greater than 0.70. We then evaluated the extent of the structure similarity information that contributed to these performances. In fact, if we do not use the SE information at all and rely only on chemical structure, only 18% of the 101 disease endpoints achieve AUCs above 0.7 (**Table S4**), while using DRoSEf 36% of disease endpoints had AUCs above 0.7. Moreover, 74% of endpoints achieved higher AUC than using chemical structural information alone. Only 22% of the variance in the AUCs of DRoSEf was explained by chemical structure across the 101 endpoints. This again indicates that the side effect intermediate is adding value to the prediction.

**Table 2:** AUCs for disease indications based on predicting side effects from structure, and then using side effects to predict the indication.

Disease category	Disease^a^	# of drugs with this indication in clinical trial	# of SE features associated with disease	AUC
**Neuropsychiatric**	Depression	72	87	0.82
	Depressive Disorder	42	204	0.82
	Schizophrenia	77	55	0.81
	Depressive Disorder, Major	48	170	0.81
	Anxiety Disorders	144	186	0.71
**Neoplasms**	Stomach Neoplasms	49	4	0.77
	Carcinoma, Non-Small-Cell Lung	73	10	0.76
	Lung Neoplasms	59	30	0.74
	Neoplasms	347	42	0.74
	Lymphoma	28	4	0.72
	Leukemia, Myeloid, Acute	30	20	0.71
	Head and Neck Neoplasms	33	7	0.70
**Others**	Hypertension	203	12	0.74
	Diabetes Mellitus, Type 2	112	8	0.71

a Only diseases with AUC>0.7 are shown.

### Case study of clinical molecules predicted to treat hypertension

**Figure 3:**
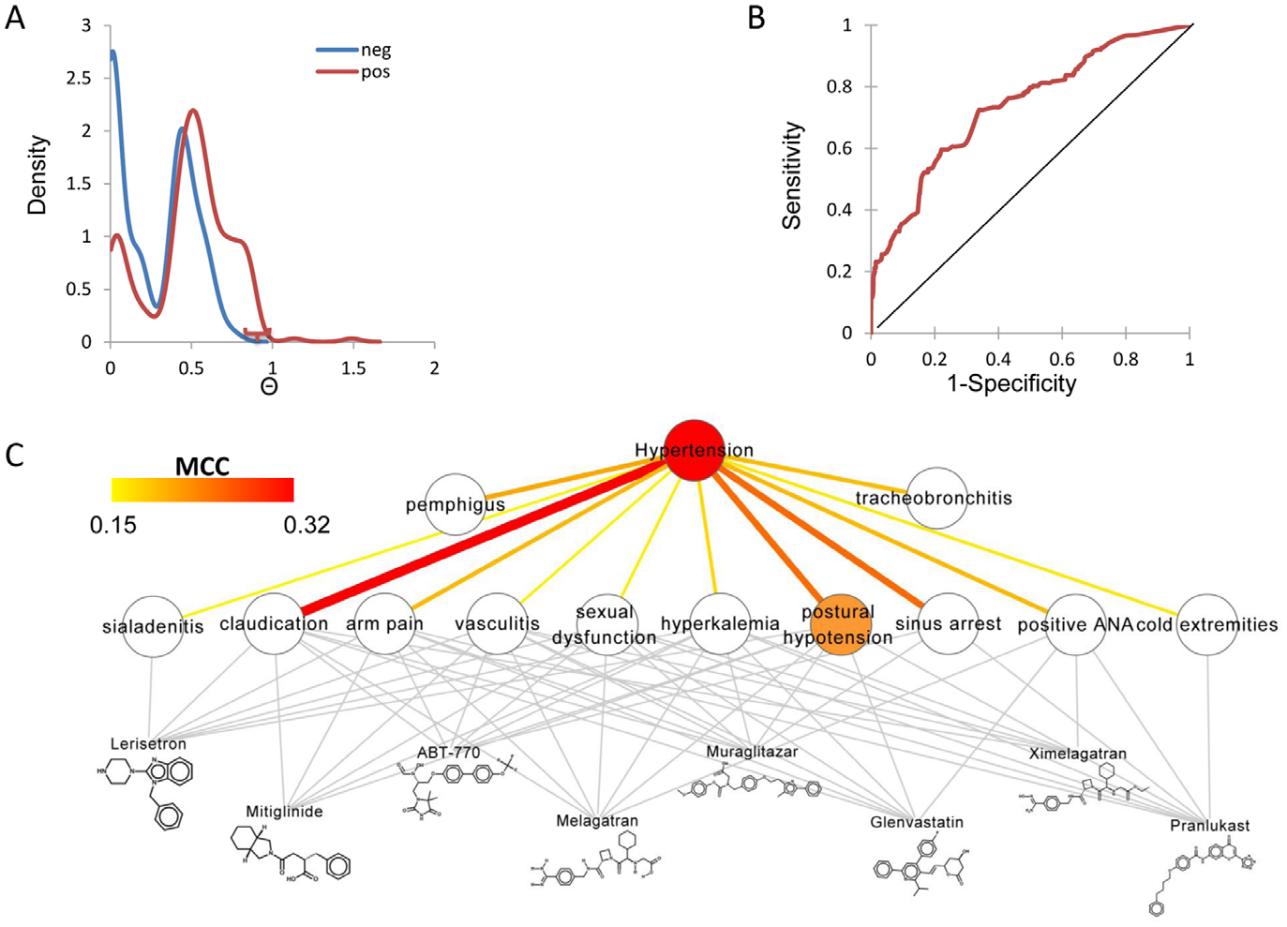
Predict drugs' repositioning potential for hypertension via DRoSEf. a) The distribution of the Θ score for the positive (red) and negative (blue) set for hypertension. The molecules with high Θ score in negative set (red square bracket) were chosen as the candidates for treating hypertension. b) The ROC curve of using Θ score to predict hypertension. The AUC is 0.74. c) Predicted relationships of the top molecules with the 12 SEs and the association of these SEs with the hypertension. The binary association among molecules and SEs is in grey lines. The association strength between SE and disease is reflected in the color and the width of the edge. *Postural hypotension* is highlighted as the SE explicitly linked to hypertension.

MetaBase includes 203 molecules indicated for hypertension. However, there are additional molecules that have not yet been reported to treat hypertension that achieved a relatively high Θ score based on SEs (corresponding to the rightmost part of the blue line in **Fig. 3A**). There are 12 SEs linked to hypertension that meet our criteria from DRoSEf. The structure of some of the molecules with the highest Θ and their predicted relationships with the 12 hypertension-associated SEs are visualized in **Fig. 3C**. Many of the SEs are physiologically linked to hypertension and the MOA for some of the SEs matched published studies. *Postural hypotension* is an obvious SE that might suggest hypertension as an indication as it is the sudden drop in blood pressure that may occur when a person stands up. Drugs causing this SE should at least be considered and evaluated for treating hypertension provided the effect can be controlled with formulation and dosing. Nine of the top 10 molecules predicted to effect hypertension from MetaBase are also predicted to induce *postural hypotension*, which is perhaps a relevant clinical phenotypic screen for hypertension and adds direct evidence for potential repositioning (**Fig. 3C**). *pemphigus* is reported to be induced by angiotensin-converting enzyme (ACE) inhibitors [42], and *cold extremities* is antihypertensives especially by β-adrenergic blockers [43]. These associations could be further confirmed in SIDER dataset, where ACE inhibitors were significantly enriched in drugs listing *pemphigus* (p = 5.7E-10) and β-blockers were associated with *cold extremities* (p = 4.5E-6). In our prediction results, we also found that ACE inhibitors are significant enriched in the drugs predicted as *pemphigus* positive (Fisher's exact p = 1.4E-3); whereas β-adrenergic blockers have significantly higher frequency in drugs predicted as *cold extremities* positive (p = 0.02). *Claudication* or peripheral artery disease, which includes narrowing and hardening of the arteries, is a SE associated with hypertension treatment. A case report demonstrated that reduction in blood pressure could worsen intermittent *claudication* [44]. *Sinus arrest* can be induced by antihypertensives [45]. ‘Sexual dysfunction’ is a known complication of some antihypertensive drug therapy and has been associated with many of the antihypertensive agents [46]. Intracranial hypotension is the cause for *arm pain* from central traction causing irritation of a cervical nerve [47]. However, we did not identify obvious MOAs for the association of vasculitis, tracheobronchitis and sialadenitis with the hypertension. In summary, the exploration of the disease-specific SEs can provide a more rational explanation for drug repositioning via understanding the known and unknown mechanism-of-action (MOA) between the SEs and the drugs' therapeutic effect.

As these are clinical molecules the amount of additional published clinical data is limited, however there is still target based validation for some of these molecules. Among the top investigational molecules in **Fig. 3C**, glenvastatin is originally indicated for hyperlipidemias. Studies have documented the effect of statins on blood pressure [48]. Melagatran and ximelagatran are thrombin inhibitors. Thrombin signaling was proved to be involved in the vascular response to hypertension [49]. Muraglitazar is an agonist of PPARα and PPARγ. PPARα stimulation exerts a lowering effect in blood pressure [50]; whereas the SEs of PPARγ agonists usually include lowering of blood pressure [51]. ABT-770 is a metalloproteinase inhibitor, and the metalloproteinase was reported to regulate blood pressure [52]. Blonanserin acts as the antagonist of 5-HT2 receptor. A study demonstrated that the increase in blood pressure is due to a stimulation of postjunctional 5-HT2 receptors [53].

## Discussion

This study proposes systematic drug repositioning based on the rational association between diseases and SEs. We extracted 3,175 relationships between diseases and SEs. For some of the drug repositioning opportunities, we found compelling published clinical trials. However, there are many new indications which have not been tested yet. We built Naïve Bayes models to predict indications for 145 diseases using the SEs as features. The AUC was above 0.8 in 92% of these models. We also extended the method to predict indications for 4,200 clinical molecules by utilizing QSAR models for SE. These results suggest that clinical pharmacologists should pay even more attention to the SEs observed in clinical trials, as they may suggest additional indications for their drugs based on understanding the connections between SEs and the therapeutic effect of the drug.

Assaf *et al* [54] systematically predicted the indication for drugs based on multiple properties of drugs and diseases. However, our focus on utilizing clinical phenotypic information enables interpretability and direct application of findings. The examples discussed in this study are primarily for demonstrating the principle of this methodology, but all of them may not necessarily be effective or practical for repositioning. Other factors need to be considered for practical use of this methodology, such as the unmet medical need for the disease, the fraction of the population showing the side effect, the CNS penetration of the molecule, and whether the therapeutic effect is significant enough in comparison to current treatments. Moreover the previous therapeutic effect could now become a potential side effect as well, and will need to be carefully considered in the risk benefit profile. But, hopefully, in a few cases this could all be managed via choosing a suitable formulation, dose, and the sub population.

The SEs have been used to predict drug targets [4]. DRoSEf mimics a phenotypic clinical assay rather than the target based assay. It has been reported that more first-in-class drugs have been found using phenotypic screening than target-based approaches between 1999 and 2008 [17]. Our study demonstrates that the clinical phenotypic features work well in suggesting new indications, and may even outperform *in vitro* assays or animal models that face many translational challenges. In this study, we did not consider the absolute frequency of the SEs or the relative frequency or significance compared to placebo.

In SIDER, only 37.9% of the drug-SE pairs have frequency information associated with them, thus to maximize the amount of drugs covered we did not utilize frequency information. SEs with higher frequencies like *nausea* and *vomiting* are usually described in detail with frequency information and written in the drug label. However, the frequencies for most of the informative SEs are unknown. Some of the SEs in **Table S1** are regarded to be rare, but are still implicated in the pathogenesis of a particular disease. In fact, they might expose an extreme phenotype. For example, *porphyria* is a rare inherited disease [55]. Patients with this inherited disease show a decrease in the risk of *porphyria* on becoming diabetic [26], [56]. This may suggest why antidiabetic drugs are usually reported to worsen *porphyria*, but this may only affect people with an inherited genetic mutation for *porphyria*, and this subset of population may in theory act as the “model” for screening anti-diabetes drugs, with *porphyria* as the screening endpoint. Thus, a drug that increases *porphyria* in this sub population with the mutation may well be a good diabetes drug in a different larger population. So the off-phenotype of a drug on a sub population might suggest its use for a broader population. In addition to mimicking a human phenotypic screen to help fish out positive candidates for repositioning, DRoSEf may also suggest the unrecognized disease pathogenesis, such as studying *porphyria* may lead to better understanding of the diabetes.

A limitation of DRoSEf is the number (888) of drugs with available side effects. The models and accuracy would improve if we were able to obtain side effects on a larger number of drugs. Moreover, predictions of indications for 4,200 MetaBase drugs would also be better if we had some side effect information from their early stage clinical trials rather than relying on just their structures. Even if we had to rely on structures for preclinical molecules, it would help if the structure based side effect models were trained on more than the 888 drugs from SIDER. The 888 molecules may not be representative in terms of structural variability, and it is possible that some of the QSAR models are over-fitted. Constructing a larger database of disease-SE associations via mining the drug labels and additional literature should improve accuracy and help reduce over-fitting. On the other hand, the prediction performance could also be an underestimate. Molecules that have not yet been reported to treat a disease may well be capable of treating that disease, and in many cases (the false positive drugs as shown in **Table 1**) clinical trials have already shown a positive effect. These molecules are classified as false positives currently, and this decreases the computed AUC value. However, even with this imperfect SE information and potentially underestimated prediction performance, 36% of the disease endpoints achieved AUCs higher than 0.7 (**Table S3**), which is generally higher than the disease prediction performance using the QSAR model alone. Although the reliability of the QSAR models needs to be considered due to the limited number of drugs in the SIDER set, the major aim of this study is not to demonstrate the power of using the QSAR model, but to emphasize that the performance of QSAR model is enhanced after incorporating side-effect information.

Using multiple SEs features to predict the disease endpoint could also improve sensitivity over individual features. Although there are explicit individual disease-SE associations, not all of them have sufficient prediction power. For instance, not all drugs treating anemia list *polycythemia* as a SE, thus the sensitivity of this feature is limited. The inclusion of multiple features could enhance sensitivity. If a true positive is not recalled by an individual feature, it may be suggested by other features. Thus better sensitivity could be achieved if we had more SEs annotations or other phenotypic terms from drug label. The emphasis on the sensitivity, however, may affect specificity. To avoid this problem, all the SEs chosen for the prediction have high specificity (sp>0.75, see **Methods**). The false positives could be excluded further through testing on *in vitro* and *in vivo* models.

DRoSEf provides numerous predictions based on the association of the SE and the disease. It greatly benefits from the fact that clinical side effects are human phenotypic data obviating translation issues. The methodology for the first time offers the possibility that the unfavorable side effects in a subpopulation can themselves offer repositioning opportunities to positively impact a broad range of patients.

## Methods

### Constructing the disease-side effect associations

The disease-SE associations were computed based on the disease-drug association and drug-SE association, which were extracted from PharmGKB and SIDER databases respectively. PharmGKB uses MeSH term to describe diseases. For side effects from SIDER, we only use them as present or absent in association with a drug, and do not consider their frequencies explicitly, as only 37.9% of the drugs had side effect frequencies associated with them. Let true positive (tp_ij_) be the number of drugs listing that are indicated for disease *i* and list *j* as a SE; false positives (fp_ij_) be the number of drugs that are not indicated for disease *i* and list SE *j*; true negatives (tn_ij_) be the number of drugs that are not indicated for disease *i* and do not list SE *j*; false negatives (fn_ij_) be the number of drugs that are indicated for *i* and do not list SE *j*. We calculated the sensitivity (sn_ij_), specificity (sp_ij_) and Matthews correlation coefficient or MCC (mcc_ij_) of using SE *j* to predict disease *i* using the standard formulas below:
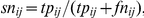


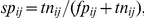


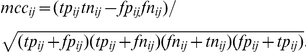
For binary variables, the MCC is the equivalent of a Pearson correlation coefficient. The two-sided Fisher's exact p_ij_ value was also calculated. A disease-SE association was considered to be non-informative, if 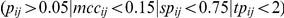
_._ This threshold provided 3,175 informative associations including 145 MeSH disease phenotypes and 584 SEs. The Fisher's p value here is a measure of the association but not an accurate estimation of the type I error as it is not corrected using the false discovery rate. The associations in **Table 1** were selected based on the following criteria: the MCC is among the top 150 of all 3,175 associations and 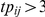
_._ From these we manually picked a few associations that had strong literature support. In **Fig. 1**, to enhance the visibility of the network layout, the disease-SE relationships were not visualized if
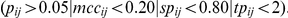



### Training the prediction models for clinical compounds

We calculated several structural descriptors (logP, molecular weight, number of hydrogen bond donors and acceptors, number of rotatable bonds and SCFP6 fingerprint) for 888 SIDER drugs. We tried to train 584 SE models with multiple Laplacian-modified Bayesian method [41] using the features above. 566 SE models were successfully trained.

### Predict the disease endpoints for clinical molecules based on SEs

We evaluated 5,534 clinical candidates and marketed drugs from Genego MetaBase (as of Jan. 2011). MetaBase uses the MeSH disease ontology for drug indications. We considered only molecules that included SMILES strings, and further listed a disease indication matching at least one of the 145 diseases from the SIDER set, and we excluded molecules that were in the 888 SIDER drug set. This left us with 4,200 small molecules. These molecules were assigned at least one of the 101 disease MeSH term that match the 145 MeSH diseases.

The endpoint of our prediction is whether or not the compound should be considered for a clinical trial for treating disease *i* just based on side effect information. For each disease *i*, we computed its side-effectome profile vector from the SIDER data,
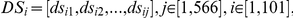
where 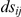 quantifies the association of disease *i* and SE *j*. The vectors were generated using seven different metrics, i.e.,
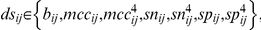
where 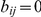 if 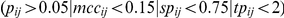
_,_ else, 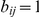
_._ We used the exponent four in an effort to enhance the signal of the high MCC, sn or sp.

For each molecule *k* without known SEs, we estimated its side-effectome profile vector 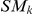 by computing it using each of the 566 pre-trained SE QSAR models,
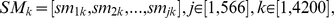
where 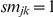 if the molecule *k* is predicted as possibly causing SE *j*, else 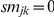
_._ We calculate the association 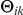 between disease *i* and molecule *k* as the dot product of the two vectors,
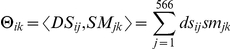
We compute 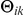 using each of the seven metrics, and for each metric we further computed an AUC for each of the 101 endpoints. The metrics 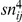 performed best among all metrics in terms of the mean AUC across all 101 disease endpoints. Thus, the AUC value in **Table S4** is based on the 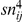 metrics.

## Supporting Information

Figure S1 **Disease-side effect network.**
(PDF)

Figure S2 **ROCs of all disease endpoints for clinical compounds.**
(TIF)

Methods S1 **Constructing the structure based prediction model for DRoSEf.**
(DOCX)

Text S1 **The hypothesis that could be made based on the connection between** ***priapism*** **and obsessive-compulsive disorder (OCD).**
(DOCX)

Table S1 **All 3,175 disease-side effect associations.**
(XLSX)

Table S2 **False positive drugs for DRoSEf.**
(XLSX)

Table S3 **AUCs for the average 10-fold cross validation of the Naïve Bayes models.**
(XLSX)

Table S4 **AUCs for 101 disease endpoints on clinical molecules, using QSAR model alone and cross validation for Naïve Bayes models.**
(XLSX)
