# Supplementary figures and images for: Systematic Drug Repositioning Based on Clinical Side-Effects
Source: PLoS One. 2011 Dec 21;6(12):e28025. doi: 10.1371/journal.pone.0028025 (PMC3244383; doi:10.1371/journal.pone.0028025)

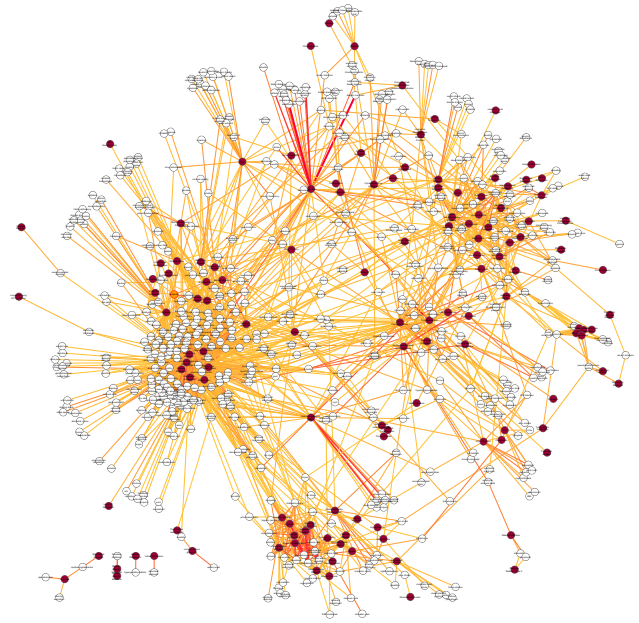

Supplement: Figure S1 — Disease-side effect network. (PDF) [file pone.0028025.s001.pdf]

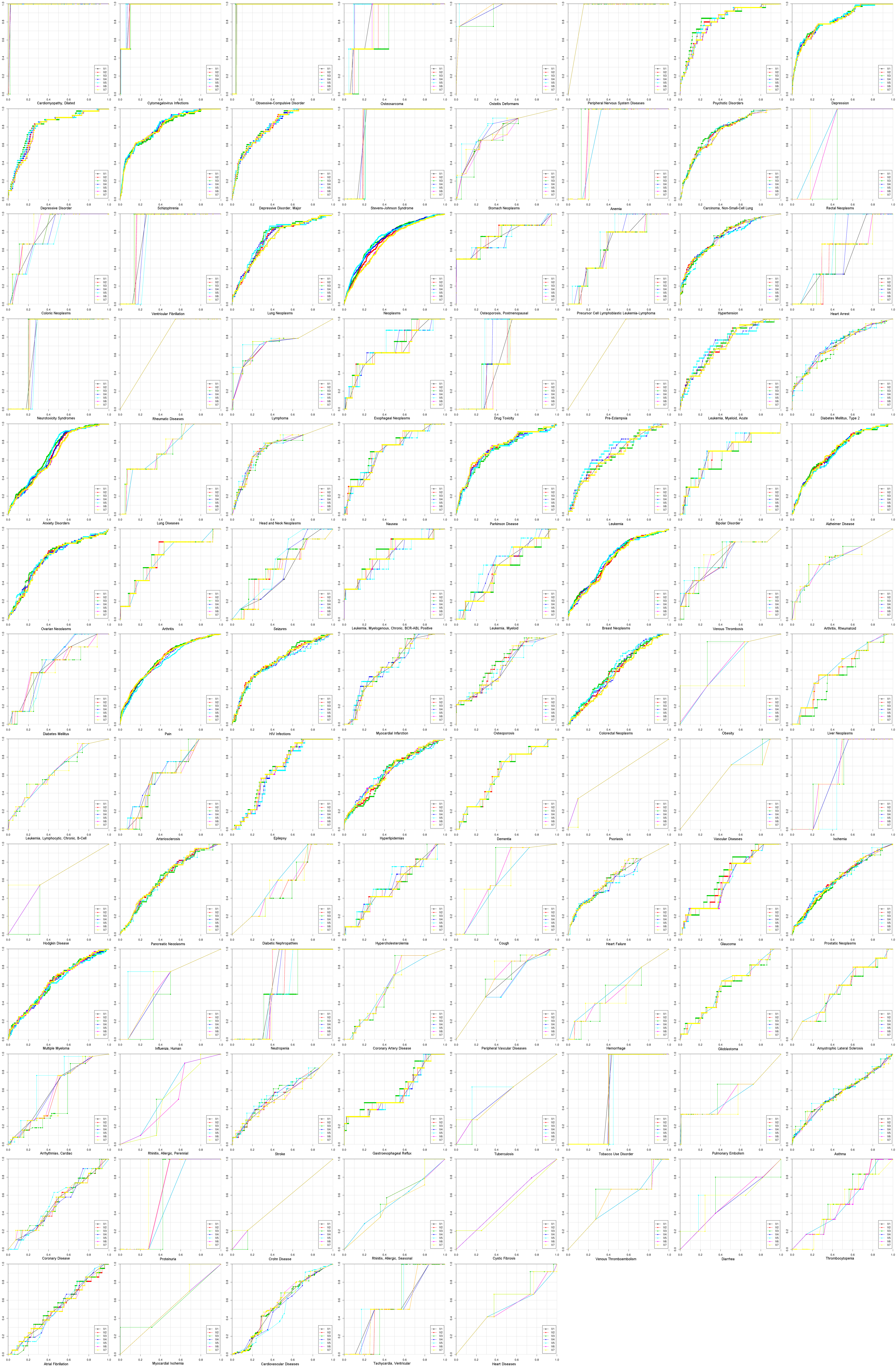

Supplement: Figure S2 — ROCs of all disease endpoints for clinical compounds. (TIF) [file pone.0028025.s002.tif]
