# Supplementary material for: Systematic Drug Repositioning Based on Clinical Side-Effects
Source: PLoS One. 2011 Dec 21;6(12):e28025. doi: 10.1371/journal.pone.0028025 (PMC3244383; doi:10.1371/journal.pone.0028025)
Supplement: Methods S1 — Constructing the structure based prediction model for DRoSEf. (DOCX) [file pone.0028025.s003.docx]

**Supplementary Methods.** Constructing the structure based prediction model for DRoSEf

Each molecule *k* could then be represented as a binary vector (SM_k_) of size 566 with position *j* being one if and only if this drug would be predicted to have side effect *j*. Each disease *i* was also independently associated with a vector (DS_i_) of 566 SEs as computed earlier based on the data from SIDER. To evaluate if molecule *k* could be used to treat disease *i*, we then sum up the 566 products for each of the elements in disease-SE (DS_i_) and the SE-molecule (SM_k_) vector. A higher score suggests that the molecule has been predicted to induce more SEs with higher association strength with the disease (**Fig. 2b**). For each disease *i*, we use the dot product value (Θ_i_) as the metric to calculate the area under curve (AUC) of the ROC.
