# Supplementary material for: Systematic Drug Repositioning Based on Clinical Side-Effects
Source: PLoS One. 2011 Dec 21;6(12):e28025. doi: 10.1371/journal.pone.0028025 (PMC3244383; doi:10.1371/journal.pone.0028025)
Supplement: Text S1 — The hypothesis that could be made based on the connection between priapism and obsessive-compulsive disorder (OCD). (DOCX) [file pone.0028025.s004.docx]

**Supporting Information 1.** The hypothesis that could be made based on the connection between *priapism* and obsessive-compulsive disorder (OCD)

First, the 7% of the drugs that are not indicated for OCD, but list *priapism* as a side effect may be potential treatments for OCD, similar to the suggestions based on the examples in Table 1. Of course one would have to consider central nervous system penetration of the drug and other biological rationale as well. Secondly, although *priapism* is regarded as an unfavorable SE during the therapies of some neuropsychiatric diseases [^1^](#_ENREF_1), it might present a repositioning opportunity with perhaps a different formulation or dose of such drugs for sexual dysfunction therapies.

Moreover, among the 7% of the drugs that have not been indicated for OCD in PharmGKB but list *priapism* as a SE, many of them have been reported to treat OCD in literature. Ziprasidone has been used as the coadjuvant treatment in resistant OCD treatment [^2^](#_ENREF_2); quetiapine was reported to be effective in treating OCD [^3-5^](#_ENREF_3); there is a case report of oxcarbazepine’s therapeutic effect in OCD [^6^](#_ENREF_6); the symptom of OCD could be decreased after olanzapine treatment [^7^](#_ENREF_7); a trend toward an non-obsessive response was seen under nefazodone treatment [^8^](#_ENREF_8); strong reduction of the OCD could be observed after using clozapine [^9^](#_ENREF_9). Noteworthy, sildenafil was also among the 7% of the drugs that cause ‘priapism’. Given the association of OCD and *priapism*, and the central nervous system penetration of sildenafil [^10^](#_ENREF_10), the drug could be considered for OCD. A possible MOA is that nitric oxide modulates the neurotransmitters implicated in OCD [^11^](#_ENREF_11), and the inhibition of PDE5 protein by sildenafil may lead to a sustained release of nitric oxide [^12^](#_ENREF_12).

**References**

1. Compton, M.T. & Miller, A.H. Priapism associated with conventional and atypical antipsychotic medications: a review. *J Clin Psychiatry* **62**, 362-366 (2001).

2. Iglesias Garcia, C., Santamarina Montila, S. & Alonso Villa, M.J. [Ziprasidone as coadjuvant treatment in resistant obsessive-compulsive disorder treatment]. *Actas Esp Psiquiatr* **34**, 277-279 (2006).

3. Alexander, J. De novo induction of obsessive-compulsive symptoms with quetiapine in a patient with borderline personality disorder. *Aust N Z J Psychiatry* **43**, 1185 (2009).

4. Vulink, N.C., Denys, D., Fluitman, S.B., Meinardi, J.C. & Westenberg, H.G. Quetiapine augments the effect of citalopram in non-refractory obsessive-compulsive disorder: a randomized, double-blind, placebo-controlled study of 76 patients. *J Clin Psychiatry* **70**, 1001-1008 (2009).

5. Savas, H.A., Yumru, M. & Ozen, M.E. Quetiapine and ziprasidone as adjuncts in treatment-resistant obsessive-compulsive disorder: a retrospective comparative study. *Clin Drug Investig* **28**, 439-442 (2008).

6. McMeekin, H. Successful treatment of obsessive compulsive disorder with oxcarbazepine. A case report. *J S C Med Assoc* **98**, 316-320 (2002).

7. van Nimwegen, L.*, et al.* Obsessive-compulsive symptoms in a randomized, double-blind study with olanzapine or risperidone in young patients with early psychosis. *J Clin Psychopharmacol* **28**, 214-218 (2008).

8. Nelson, E.C. An open-label study of nefazodone in the treatment of depression with and without comorbid obsessive compulsive disorder. *Ann Clin Psychiatry* **6**, 249-253 (1994).

9. Peters, B. & de Haan, L. Remission of schizophrenia psychosis and strong reduction of obsessive-compulsive disorder after adding clozapine to aripiprazole. *Prog Neuropsychopharmacol Biol Psychiatry* **33**, 1576-1577 (2009).

10. Schultheiss, D.*, et al.* Central effects of sildenafil (Viagra) on auditory selective attention and verbal recognition memory in humans: a study with event-related brain potentials. *World J Urol* **19**, 46-50 (2001).

11. Umathe, S.N.*, et al.* Role of nitric oxide in obsessive-compulsive behavior and its involvement in the anti-compulsive effect of paroxetine in mice. *Nitric Oxide* **21**, 140-147 (2009).

12. Ghofrani, H.A., Osterloh, I.H. & Grimminger, F. Sildenafil: from angina to erectile dysfunction to pulmonary hypertension and beyond. *Nat Rev Drug Discov* **5**, 689-702 (2006).
